# Antimicrobial Chemotherapy for Recalcitrant Severe Human Periodontitis

**DOI:** 10.3390/antibiotics12020265

**Published:** 2023-01-28

**Authors:** Thomas E. Rams, Jørgen Slots

**Affiliations:** 1 Department of Periodontology and Oral Implantology, Temple University School of Dentistry, Philadelphia, PA 19140, USA; 2 Division of Periodontology and Diagnostic Sciences, University of Southern California School of Dentistry, Los Angeles, CA 90089, USA

**Keywords:** anti-infective agents, periodontitis, periodontal pathogens, metronidazole, ciprofloxacin, amoxicillin, povidone-iodine, sodium hypochlorite

## Abstract

This study evaluated a combined systemic and topical anti-infective periodontal treatment of 35 adults who had experienced ongoing periodontal breakdown following conventional surgical periodontics. The prescribed anti-infective therapy, based on microbiological testing, consisted of a single course of metronidazole plus ciprofloxacin (23 patients), metronidazole plus amoxicillin/clavulanic acid (10 patients), and metronidazole plus ciprofloxacin followed by metronidazole plus amoxicillin/clavulanic acid (2 patients). In addition, the study patients received 0.1% povidone-iodine subgingival disinfection during non-surgical root debridement and daily patient administered oral irrigation with 0.1% sodium hypochlorite. At 1 and 5 years post-treatment, all study patients showed gains in clinical periodontal attachment with no further attachment loss, and significant decreases in pocket probing depth, bleeding on probing, and subgingival temperature. The greatest disease resolution occurred in patients who at baseline harbored predominantly major periodontal pathogens which post-antibiotics became non-detectable and substituted by non-periodontopathic viridans streptococci. The personalized and minimally invasive anti-infective treatment regimen described here controlled periodontitis disease activity and markedly improved the clinical and microbiological status of the refractory periodontitis patients.

## 1. Introduction

Human periodontitis involves interaction between active (lytic) herpesviruses, bacterial pathogens, and destructive pro-inflammatory host immune responses [1,2]. Potential periodontopathic bacteria include anaerobic Gram-negative rods (*Porphyromonas gingivalis*, *Tannerella forsythia*, *Prevotella intermedia/nigrescens*, *Fusobacterium* species, *Selenomonas noxia*, and *Fretibacterium fastidiosum*), some spirochetal *Treponema* species, anaerobic Gram-positive cocci (*Parvimonas micra*) and rods (*Eubacterium nodatum* and *Filifactor alocis)*, facultative Gram-positive cocci (*Streptococcus constellatus*, staphylococci, and enterococci), the Gram-negative capnophilic rod *Aggregatibacter actinomycetemcomitans*, the Gram-negative microaerophilic motile rod *Campylobacter rectus*, Gram-negative facultative enteric rods, pseudomonads and related organisms, and perhaps the yeast *Candida albicans* in some patients [3,4,5]. A number of microbial phylotypes yet to be cultivated and studied for virulence factors have also been statistically associated with periodontitis lesions [5]. In contrast, subgingival bacterial communities after clinically successful periodontal therapy predominantly contain facultative Gram-positive streptococci (*Streptococcus sanguinis*, *Streptococcus mitis*, and *Streptococcus oralis*), *Actinomyces, Rothia* and *Corynebacterium* species, facultative Gram-negative *Capnocytophaga* species and *Lautropia mirabilis*; and anaerobic Gram-negative *Veillonella* species [4,6,7]. 

The subgingival microbial composition is an important determinant of the efficacy of periodontal therapy. Mechanical root instrumentation and periodontal surgery alone cannot eradicate *A*. *actinomycetemcomitans*, black-pigmented anaerobic rods, *T. forsythia*, *P*. *micra*, and several of the enteric organisms from deep periodontal pockets or invaded periodontal tissues [8,9,10,11,12,13,14,15,16,17,18,19,20]. Individuals populated by these bacteria tend to belong to the 10–15% of periodontitis patients who are “refractory” to conventional periodontal therapy [6,21]. In contrast, an appropriate adjunctive systemic antimicrobial therapy has the potential to markedly suppress or even eradicate persistent subgingival and tissue-invading pathogens [22,23]. However, overgrowth of pathogen(s) and aggravated types of periodontitis may result if the targeted pathogens are resistant to the prescribed antibiotics [24].

Metronidazole alone is effective against various periodontal anaerobes [25] but is ineffective against facultative organisms such as *A*. *actinomycetemcomitans*, *S. constellatus*, enteric rods, pseudomonads, staphylococci, and enterococci [26,27,28,29,30,31]. Broad-spectrum single-drug tetracycline family antibiotics demonstrate large person-to-person variation in gastrointestinal absorption [32] and effectiveness [22,23,33], and may predispose patients to opportunistic periodontal superinfections by resistant organisms [34].

Poorly defined criteria for drug selection are a major reason for inappropriate and ineffective use of systemic antimicrobials in periodontics. Important prerequisites for selecting a systemic antimicrobial therapy in periodontics are (1) the antimicrobial agent should exhibit inhibitory in vitro concentrations that are achievable in vivo, (2) in vivo evidence that the antimicrobial therapy can eradicate or markedly suppress subgingival and tissue-invading putative bacterial pathogens, (3) the antimicrobial therapy should not result in the establishment of superinfecting pathogens, (4) the post-treatment microbiota should be predominated by organisms compatible with periodontal health, (5) the antimicrobial agent should not give rise to resistant microfloras which could compromise future dental or medical usage of the drugs, (6) the therapy should have no or minimal adverse medical side effects, and (7) the antimicrobial agent should demonstrate little or no interactions with other patient medications [35].

Antibiotics in combination, particularly metronidazole plus ciprofloxacin, seem to fulfill these requirements. Metronidazole affects the anaerobic segment of the periodontal microflora [25], and ciprofloxacin is highly active against many facultative pathogens, including *A. actinomycetemcomitans*, *Eikenella corrodens*, enteric rods, pseudomonads, staphylococci and enterococci [22,23,26,28,29,30,31,35,36,37,38,39]. The two drugs in combination have been employed successfully against mixed facultative/anaerobic infections at other body sites [40,41,42,43,44], are not antagonists [45,46], and may even act synergistically against some bacterial species [47,48], including periodontal *A*. *actinomycetemcomitans* [49]. Furthermore, in vitro data indicate that “beneficial” streptococci may constitute a major proportion of the subgingival microflora following metronidazole plus ciprofloxacin therapy [37].

Combinations of metronidazole plus amoxicillin or amoxicillin/clavulanic acid [35] also show synergistic activity against periodontal *A*. *actinomycetemcomitans* [50] and in vivo suppresses *P*. *gingivalis*, *T. forsythia*, *Treponema denticola*, *Eubacterium nodatum* and a number of other putative periodontal pathogens [51,52,53].

The present case-series study evaluated the clinical and microbiological efficacy of a combined anti-infective periodontal therapy composed of conventional non-surgical mechanical root debridement augmented with locally applied antiseptic agents and short-term systemic antibiotics. The antibiotic regimens employed included combinations of metronidazole, ciprofloxacin and amoxicillin/clavulanic acid, chosen on the basis of the microbiological composition of the patient’s subgingival microbiota. A total of 35 adults with a history of disease-active periodontitis following mechanical-surgical periodontal therapy were studied. The relationship between the post-treatment microbial composition and the periodontal disease resolution was evaluated as well.

## 2. Materials and Methods

### 2.1. Patients

The study patients were identified from consecutive de-identified patient records at a private periodontal specialty practice in Washington, DC, USA. All diagnostic and treatment procedures were performed by a single experienced board-certified periodontist (author T.E.R.). A total of 35 systemically healthy adults (17 males and 18 females, aged 36–70 years, mean age 50.3 years, 32 white, 1 black, 2 Asian) were diagnosed with localized to generalized Stage III/Grade C severe periodontitis [54]. Two (5.7%) of the patients were current smokers. 

Inclusion criteria for patients in the case-series were as follows:

(1). Ongoing periodontal attachment loss and radiographic bone loss on ≥3 teeth despite comprehensive periodontal therapy provided during the previous 1–4 years by periodontists prior to referral to author T.E.R. The previous periodontal therapy included repeated subgingival debridement, surgical flap procedures, oral hygiene instructions, and systematic maintenance care. Eight patients developed periodontal abscesses despite these treatment procedures and were empirically prescribed ≥1 systemic regimens of tetracycline and/or penicillin antibiotics.
(2). A generalized absence of crestal lamina dura [55,56] at interproximal tooth sites on radiographs taken 1–4 years after the previous periodontal therapy.
(3). Persistence, despite previous periodontal therapy, of a high-risk subgingival microbiota associated with progression of periodontitis [57]. This was defined as cultivable detection of either *A. actinomycetemcomitans* and/or *P. gingivalis*, and/or cultivable recovery of *P. intermedia/nigrescens* at ≥2.5%, *P. micra* at ≥3.0%, *C. rectus* at ≥2.0%, and/or either Gram-negative enteric rods/pseudomonads, staphylococci, or *Candida* species at ≥5.0%, of total subgingival viable counts.
(4). Minimal or no supragingival dental plaque or clinically detectable subgingival dental calculus because of previous periodontal therapy.
(5). No medical history precluding exposure to povidone-iodine, metronidazole, ciprofloxacin, or amoxicillin/clavulanic acid [22,58], except for 6 patients with a history of hypersensitivity reactions to penicillin antibiotics.
(6). Patient informed consent for periodontal therapy in compliance with the Helsinki Declaration of 1975, as revised in 2000.
(7). Post-treatment outcomes documented at 1 month, 1 year, and 5 years post-treatment.

The Temple University Human Subjects Institutional Review Board reviewed this practice-based retrospective study and determined that no action was applicable on their part, since no investigator–patient contact, interaction, or intervention was involved beyond analysis of archived dental practice data not linked to the identification of any person(s).

### 2.2. Clinical Evaluations

Periodontal probing depth, clinical periodontal attachment level from the cementoenamel junction or restoration margins on tooth surfaces, and gingival bleeding after probing within 30 s were measured at 6 sites on all teeth in each patient using a computer-linked Florida Probe system (Florida Probe Corporation, Gainesville, FL, USA) with a 0.4 mm diameter periodontal probe tip and a constant probing force of 0.196 newtons (20 g) [59]. Periodontal sites sampled for microbiological analysis were separately assessed with the Plaque Index on a 0 to 3 scale [60]. Clinical evaluations at 1 and 5 years post-treatment were performed blindly without access to baseline data. 

At 1 year post-treatment, subgingival temperature at microbiologically sampled periodontal sites, as well as reference sublingual body core temperature from under the most posterior and medial part of the tongue, were measured in each patient to the nearest 0.01 °C using a PerioTemp Probe System (ABIODENT, Inc., Danvers, MA, USA) [61]. To account for variation in body core temperature between patients, sublingual temperature was subtracted from subgingival temperatures in each patient, providing a temperature differential between subgingival and body core temperature for each evaluated periodontal site. A software algorithm in the temperature probe’s internal computer then compared each temperature differential to anatomically specific subgingival temperature reference values found at periodontally healthy sites [62], and classified the evaluated periodontal sites as either hot, intermediate, or cool, as indicated by illumination of red, yellow, or green light-emitting diodes, respectively, on the instrument console. A temperature indicator score of 1 was assigned for green lights (indicating cool subgingival temperatures), 2 for yellow lights (intermediate subgingival temperatures), and 3 for red lights (hot subgingival temperatures) [63].

The periodontist who clinically evaluated the patients (author T.E.R.) demonstrated a high degree of intra-examiner reproducibility in measurements of probing depth, clinical periodontal attachment level, subgingival temperature, and sublingual temperature. Replicate Florida Probe measurements of 490 periodontal sites with <5 mm probing depths and 102 periodontal sites with a ≥5 mm probing depth revealed mean differences of only 0.03 mm (2.66 mm versus 2.69 mm) and 0.09 mm (5.54 mm versus 5.63 mm), respectively, between duplicate probing depth measurements of more shallow (<5 mm) and deeper (≥5 mm) sites, with standard deviations for the differences between the replicate measurements to be only 0.31 mm and 0.59 mm, respectively [59]. For clinical periodontal attachment level, 100% agreement within 2 mm was attained with replicate assessments on 336 periodontal sites in 4 adults with periodontitis. Replicate measurements of 91 sulcular and 9 sublingual temperatures yielded mean temperature differences of only 0.10 ± 0.15 (SD) °C and 0.04 ± 0.04 (SD) °C, respectively [61]. 

### 2.3. Microbiological Testing

Subgingival microbial specimens were collected from 4–6 deep (≥6 mm) periodontal pockets with bleeding on probing in each patient at baseline and at 1 and 5 years post-treatment. The sample sites were isolated with cotton rolls, followed by removal of saliva/supragingival plaque and insertion of 1–2 sterile absorbent paper points (Johnson & Johnson, East Windsor, NJ, USA) into the depth of the pockets for 10 s. The paper points from each patient were then pooled into a glass vial containing an anaerobically prepared and stored transport medium (VMGA III) specifically designed for oral microorganisms [64]. The sample vials were maintained at room temperature and transported within 24 h to the Oral Microbiology Testing Laboratory, University of Southern California School of Dentistry, Los Angeles, CA, USA, which was licensed for high-complexity bacteriologic analysis by the California Department of Public Health, and federally certified by the United States Centers for Medicare and Medicaid Services to be in compliance with Clinical Laboratory Improvement Amendments (CLIA) regulations on diagnostic testing of human specimens [65]. All laboratory procedures were performed independently by personnel who were unaware of the clinical status of the patients and their inclusion in the present study.

At the laboratory, the specimen vials were warmed to 35 °C to liquefy the VMGA III transport medium, and sampled microorganisms were mechanically dispersed from paper points with a vortex mixer set at the maximal setting for 45 s. Serial 10-fold dilutions of bacterial suspensions were prepared in Möller’s VMGA I anaerobic dispersion solution [66], with 0.1 mL dilution aliquots spread with a sterile bent-glass rod onto (1) nonselective enriched Brucella blood agar (EBBA) primary isolation plates [67], composed of 4.3% Brucella agar supplemented with 0.3% bacto-agar, 5% defibrinated sheep blood, 0.2% hemolyzed sheep red blood cells, 0.0005% hemin, and 0.00005% menadione, to recover total viable subgingival counts, *P. gingivalis*, *P. intermedia*/*nigrescens*, *P. micra*, staphylococci, and *Enterococcus faecalis*, (2) TSBV agar [68] to quantitate *A. actinomycetemcomitans*, Gram-negative enteric rods/pseudomonads, and yeasts, (3) Hammond’s selective medium to identify *C. rectus* [69], and (4) mitis-salivarius agar (Difco Laboratories, Detroit, MI, USA) supplemented with 0.1% potassium tellurite (Difco Laboratories) for isolation of non-periodontopathic *Streptococcus sanguinis* and *Streptococcus mitis* subgroups. EBBA plates and the selective medium for *C. rectus* were incubated at 35 °C for 10 days in a Coy anaerobic chamber (Coy Laboratory Products, Ann Arbor, MI, USA) containing 85% N_2_-10% H_2_-5% CO_2_, and the TSBV plates at 35 °C for 3 days in 10% CO_2_-90% air. Mitis-salivarius plates were incubated anaerobically at 35 °C for 2 days, followed by aerobic incubation at room temperature for 24 h. 

Periodontal pathogens, superinfecting species and viridans streptocoocci were presumptively identified followed established criteria and techniques [57,68,69,70,71,72,73]. The proportional recovery of each evaluated species was calculated per patient as the percentage of species colony-forming units relative to total viable subgingival anaerobic counts.

For in vitro antibiotic susceptibility testing, 0.1 mL aliquots of baseline subgingival sample dilutions were inoculated onto EBBA primary isolation plates supplemented with 1.0 mg/L of either amoxicillin, metronidazole, ciprofloxacin, or a combination of metronidazole plus ciprofloxacin (all obtained from Sigma Chemical Company, St. Louis, MO, USA), and incubated anaerobically at 35 °C for 10 days, as previously described [37]. Antibiotic activity against the test microbial species was assessed by comparing growth on nonselective and antibiotic-supplemented EBBA isolation plates [38].

### 2.4. Clinical Treatment Protocol 

Each patient was initially treated with an air polishing instrument (EMS Air-Flow S1, Electro Medical Systems, Le Sentier, Switzerland) with a sodium bicarbonate-based prophylaxis powder-water-air mixture (EMS Air-Flow Classic, Electro Medical Systems; 65 μm median particle grain size) to disrupt supra- and subgingival tooth biofilms, as described elsewhere [74]. This was followed by thorough periodontal root debridement with hand and ultrasonic (Piezon Master 400, Electro Medical Systems, Le Sentier, Switzerland) scaling instruments performed under local anesthesia in 1–2 clinic sessions until smooth, hard root surfaces devoid of clinically detectable subgingival calculus deposits were obtained, as determined with an ODU 11/12 dental explorer (Hu-Friedy Manufacturing Company, Chicago, IL, USA) [75,76]. A freshly-mixed 0.1% povidone-iodine solution (Povidone-Iodine 10% Antiseptic Solution^-^, Rite Aid Corporation, Camp Hill, PA, USA; diluted one part to 99 parts water), providing approximately 25 ppm free iodine [77], was used as the ultrasonic scaler coolant and continuously introduced into subgingival sites during ultrasonic scaler instrumentation [78]. High-volume evacuation was used during ultrasonic scaling to minimize patient absorption/ingestion of povidone-iodine.

Prescribed patient home oral hygiene procedures included twice daily toothbrushing with a 65% sodium bicarbonate fluoridated dentifrice (Arm & Hammer Dental Care Toothpaste, Church & Dwight Company, Ewing Township, NJ, USA), flossing and/or interproximal use of an interdental brush or rubber cone stimulator (GUM^®^, Sunstar Americas, Inc., Schaumburg, IL, USA), and daily use of an oral irrigation device (Waterpik^®^, Water Pik, Inc., Fort Collins, CO, USA) at a moderate to high pressure setting with a blunt-ended classic jet irrigator tip [79]. A freshly-mixed 0.1% diluted sodium hypochlorite (household bleach) solution was recommended to be intraorally applied with the oral irrigator (obtained by mixing one teaspoon of 6% Clorox Regular Bleach (Clorox Company, Oakland, CA, USA) into 10 fluid ounces of tap water). Emphasis was placed on patient delivery of diluted sodium hypochlorite into interproximal areas. Patients were advised to rinse with tap water after oral irrigator use. At baseline and post-treatment appointments, patients were shown phase-contrast microscopic projections of their subgingival morphotypes to aid in patient education and motivation, and to assess patient compliance with prescribed home care procedures [79,80,81]. 

After completion of local periodontal therapy and establishment of anti-infective patient home care, a short-term systemic antibiotic regimen was prescribed based on the composition of the subgingival microbiota and the medical history of each patient. A total of 23 patients received an 8-day course of systemic metronidazole plus ciprofloxacin (500 mg of each medication orally every 12 h per day) (Treatment Group I). These individuals showed a baseline subgingival microbiota with elevated proportions of both anaerobic and facultative periodontal pathogens which were susceptible in vitro to one or both of the prescribed antibiotics. Among these patients, 17 harbored Gram-negative enteric rods/pseudomonads that were susceptible to ciprofloxacin but resistant to metronidazole and amoxicillin, and 6 patients were positive for *A. actinomycetemcomitans* but hypersensitive to penicillin antibiotics. 

Another 10 patients without a penicillin allergy history were prescribed an 8-day course of systemic metronidazole plus amoxicillin/clavulanic acid (Augmentin^®^, SmithKline Beecham Pharmaceutical, Philadelphia, PA, USA) (250 mg of each medication orally every 8 h per day) (Treatment Group II). These patients revealed at baseline subgingival *A. actinomycetemcomitans* or a mixed group of anaerobic and facultative periodontal pathogens which demonstrated in vitro susceptibility to one or both of the antibiotics. 

An additional 2 patients were initially prescribed a 4-day course of systemic metronidazole plus ciprofloxacin (500 mg of each medication orally every 12 h per day), followed by a 4-day course of metronidazole plus amoxicillin/clavulanic acid (250 mg of each medication orally every 8 h per day) (Treatment Group III). These 2 patients yielded a baseline subgingival microbiota composed of *A. actinomycetemcomitans*, ciprofloxacin-susceptible *Pseudomonas* species, and several species of anaerobic periodontal pathogens, including metronidazole-resistant and ciprofloxacin-resistant strains of *P. micra*, which were sensitive in vitro to amoxicillin. All periodontal pathogens recovered in the 2 patients were susceptible in vitro to one or more of the prescribed antibiotics. 

The patients were contacted during their systemic drug regimens to monitor compliance and adverse side effects. Medication intake was confirmed by an absence or marked decrease of spirochetal morphotypes in wet-mount preparations of subgingival specimens examined with phase-contrast microscopy [82]. Supportive periodontal therapy was carried out at 3–4 month intervals in a single clinic session and included baseline anti-infective topical procedures.

**Figure 1:**
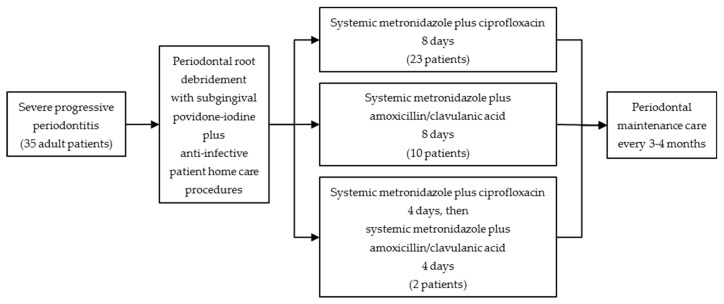
Flow chart of the study protocol.

Figure 1 summarizes the study protocol.

### 2.5. Data Analysis

Clinical periodontal parameters were evaluated at baseline and at 1 and 5 years post-treatment, and the microbiological variables at baseline, 1 month, and 1 year post-treatment. Means and standard deviation (SD) were calculated for continuous variables, and frequencies and percentages for categorical variables. Each patient was assessed for the number of teeth exhibiting probing depths ≥6 mm, which are at increased risk of post-treatment periodontitis progression [83], and for mean Plaque Index scores, mean subgingival temperature/indicator scores, and the percentage of periodontal sites exhibiting bleeding on probing. The number of periodontal sites per patient with a probing depth ≥5 mm plus bleeding on probing was also determined as an indicator of further periodontal treatment need [84] and risk for post-treatment patient tooth loss [83]. The percentage of periodontal sites per patient exhibiting a post-treatment gain or loss of clinical periodontal attachment >2 mm, a threshold considered unlikely to be explained by examiner measurement error [85], was enumerated as an outcome variable that is clinically more meaningful than mean whole-mouth changes in clinical attachment level [86,87]. 

Changes in periodontopathic species were determined by summing periodontal pathogens and superinfecting species for each patient, and then calculating total mean values across all patients, as previously described [20]. The nonparametric Wilcoxon matched pairs signed-rank test and McNemar’s non-parametric test with Yates’ continuity correction assessed clinical and microbiological changes from baseline. The Student’s t-test evaluated the relationship between post-treatment persistence of a high-risk periodontopathic microbiota [57] and the mean number of periodontal sites per patient with post-treatment probing depth ≥5 mm plus bleeding on probing. A *p*-value of ≤0.05 was required for statistical significance. Data analysis was performed using a 64-bit statistical software package (STATA/SE 16.0 for Windows, StataCorp PL, College Station, TX, USA).

## 3. Results

### 3.1. Clinical Outcomes 

Table 1 presents baseline and post-treatment clinical periodontal parameters in the three patient treatment groups. 

The study patients had a mean 26.9 ± 2.8 (SD) teeth at baseline. Patients in each treatment group averaged 29.6–40.8% teeth with probing depths ≥6 mm at baseline, and exhibited widespread gingival bleeding on probing (mean 21.2–48.5% of periodontal sites) (Table 1). 

After treatment, Plaque Index scores tended to be lower in the patient treatment groups at 1 and 5 years post-treatment, but were not statistically different from the relatively low baseline values (Table 1). A total of 12 teeth in 10 patients were lost over the 5 year post-treatment period as follows: 1 tooth in each of 4 patients due to persistent severe tooth mobility (>2 mm horizontal movement) present at baseline; 5 teeth in 3 patients due to unrestorable root caries; 1 tooth in each of 2 patients due to fracture; and 1 tooth for orthodontic treatment reasons. All lost teeth had baseline periodontal probing depths ≤4 mm. 

Among patients treated with systemic metronidazole plus ciprofloxacin (Treatment Group I), statistically significant decreases from baseline values at 1 and 5 years post-treatment were found in the percentage of teeth per patient with probing depths ≥6 mm (3.4-fold and 4.1-fold decreases, respectively), the percentage of periodontal sites per patient with gingival bleeding on probing (12.2-fold and 14.5-fold decreases), and the number of periodontal sites per patient with both probing depths ≥5 mm and bleeding on probing (23.8-fold and 27.8-fold decreases) (Table 1). At 5 years post-treatment, only 2.1% of sites per patient exhibited bleeding on probing, and only 1.2 sites per patient showed ≥5 mm probing depth with bleeding on probing. Gains of clinical attachment >2 mm were measured on average of 4.8% of periodontal sites per patient at 1 year post-treatment, and at 5.9% of sites at 5 years post-treatment. No additional loss of clinical attachment >2 mm occurred at any periodontal site in Treatment Group I patients at both 1 and 5 years post-treatment (Table 1). Cool subgingival temperatures were measured in the patients at 1 year post-treatment, as indicated by mean temperature indicator scores of 1.3 per patient, and mean temperature differentials of −0.4 °C per patient between subgingival and sublingual body core temperature measurements (Table 1).

Similar probing depth reductions, decreased bleeding on probing, gains of clinical attachment, and cool subgingival temperatures also occurred in Treatment Group II and III patients, and no additional loss of clinical attachment >2 mm was detected in any of patients at 1 and 5 years post-treatment (Table 1). In patients treated with systemic metronidazole plus amoxicillin/clavulanic acid (Treatment Group II), statistically significant decreases were found at 1 and 5 years post-treatment in probing depths ≥6 mm (3.3-fold and 3.2-fold decreases, respectively), gingival bleeding on probing (7.9-fold and 12.5-fold decreases), and probing depths ≥5 mm with bleeding on probing (14.5-fold and 11.6-fold decreases) (Table 1). At 5 years post-treatment, bleeding on probing was present at only 1.7% of sites per patient in Treatment Group II patients, with 2.5 sites per patient revealing a ≥5 mm probing depth with bleeding on probing. Gains of clinical attachment >2 mm were detected at a mean 10.2% of periodontal sites per patient at 1 year post-treatment, and at 6.3% of sites at 5 years post-treatment. 

The 2 patients in Treatment Group III had all probing depths <6 mm at 1 and 5 years post-treatment, along with marked decreases in both bleeding on probing (28.5-fold at 1 year and 13.9-fold at 5 years post-treatment), and probing depths ≥5 mm with bleeding on probing (44.0-fold decreases at both post-treatment time points) (Table 1). At 5 years post-treatment, bleeding on probing was detected at a mean of 3.5% of sites per patient, with only an average of 0.5 sites per patient demonstrating a ≥5 mm probing depth with bleeding on probing. Gains of clinical attachment >2 mm were found at an average of 9.9% of periodontal sites per patient at 1 year post-treatment, and at 6.8% of sites at 5 years post-treatment.

No adverse side effects (i.e., increased tooth root sensitivity, gingival/oral mucosal tissue abrasions, subcutaneous air emphysema, post-treatment periodontal abscesses) were observed or reported by patients in any of the treatment groups following the locally applied periodontal therapy (air polishing, periodontal root debridement, povidone-iodine subgingival delivery, home oral hygiene procedures). During systemic periodontal antibiotic therapy, 14 (60.9%) patients in Treatment Group I reported no adverse side effects, 7 (30.4%) experienced intermittent loose stools/nausea, and 3 (13.0%) noted a transient metallic taste in their saliva. For patients in Treatment Group II, six (60.0%) reported no adverse side effects, and four (40.0%) experienced intermittent loose stools/nausea during the systemic antibiotic course. Both patients in Treatment Group III reported no systemic antibiotic-related adverse effects.

### 3.2. Microbiological Outcomes 

Table 2 presents microbial changes from baseline in Treatment Group I. 

A total of 17 Treatment Group I patients at baseline yielded high subgingival levels of enteric rods/pseudomonads (mean 20.3% of cultivable subgingival counts) and often *C. rectus*, *P. micra*, and *P. intermedia/nigrescens*. *Aggregatibacter actinomycetemcomitans* was recovered from six patients and *P. gingivalis* from five patients prior to treatment. At 1 month post-treatment, *A. actinomycetemcomitans* and *P. gingivalis* were not detected in Treatment Group I patients, and marked reductions occurred in the subgingival prevalence and cultivable proportions of enteric rods/pseudomonads, *P. intermedia/nigrescens*, *P. micra*, and *C. rectus* (Table 2). Total proportions of test periodontal pathogens and superinfecting species significantly decreased from a mean of 22.9% to 0.9% per patient, and the number of patients exhibiting a high-risk subgingival microbiota significantly decreased from 23 (100%) to 2 (13%) patients (Table 2). Subgingival proportions of viridans streptococci also significantly increased from an average 2.6% to 24.2% per patient. Similar subgingival changes from baseline were found at 1 year post-treatment in Treatment Group I patients (Table 2).

Table 3 reveals microbial changes from baseline in Treatment Group II. 

At baseline, most of the patients in Treatment Group II had elevated subgingival levels of *A. actinomycetemcomitans*, *P. intermedia/nigrescens*, *P. micra*, and *C. rectus*. At 1 month post-treatment, *A. actinomycetemcomitans*, *P. gingivalis*, and *P. intermedia/nigrescens* were not detected in Treatment Group II patients, and *P. micra* and *C. rectus* were markedly reduced in their subgingival presence and cultivable proportions (Table 3). Total cultivable proportions of test periodontal pathogens and superinfecting species decreased significantly from an average of 18.2% to 1.3% per patient. However, one patient yielded 37.5% *Enterobacter cloacae* and 3.5% *P. micra*, and another had 6.9% *P. micra*, leaving two (20%) of the Treatment Group II patients with a high-risk subgingival microbiota at 1 month post-treatment (Table 3). At 1 year post-treatment, this increased to four (40%) of the patients in Treatment Group II, largely due to recolonization by *A. actinomycetemcomitans* or increased levels of *P. micra*, *P. intermedia/nigrescens*, and/or *C. rectus*. Subgingival levels of viridans streptococci significantly increased from a mean 5.7% per patient at baseline to 18.5% at 1 month and to 19.1% at 1 year post-treatment.

Table 4 details microbial changes from baseline in Treatment Group III. 

At baseline, both patients in Treatment Group III showed high subgingival levels of *A. actinomycetemcomitans*, *P. intermedia/nigrescens*, *P. micra*, *C. rectus*, and enteric rods/pseudomonads. At 1 month post-treatment, *A. actinomycetemcomitans*, *P. intermedia/nigrescens*, and enteric rods/pseudomonads were not detected in both patients, and levels of *P. micra* and *C. rectus* were markedly reduced (Table 4). Total proportions of the test periodontal pathogens and superinfecting species were significantly decreased from a mean 50.7% to 0.9% per patient, and neither of the two patients exhibited a high-risk post-treatment subgingival microbiota. A similar microbiological status was also found at 1 year post-treatment (Table 4). Subgingival levels of viridans streptococci significantly increased from an average 6.5% at baseline to 16.6% at 1 month and 31.0% at 1 year post-treatment. 

Table 5 examines among all 35 study patients the post-treatment relationship between a high-risk subgingival microbiota associated with periodontitis progression and clinical periodontal disease resolution.

A total of 12 patients yielded a high-risk subgingival microbiota at 1 month and 1 year post-treatment, and 23 patients revealed an absence of a high-risk subgingival microbiota at both time points. The 12 patients with a continued post-treatment presence of a high-risk subgingival microbiota had a significantly greater number of periodontal sites with persisting probing depths ≥5 mm plus bleeding-on-probing at both 1 year (mean 3.2 vs. 0.7 sites; 4.6-fold higher) and 5 years post-treatment (2.9 vs. 0.8 sites; 3.6-fold higher) than patients negative post-treatment for a high-risk subgingival microbiota. 

## 4. Discussion 

The primary goal of periodontitis treatment is the control or eradication of major pathogens, and establishment of a health-associated microbiome [88]. Contemporary periodontics considers both specific bacteria and active herpesviruses as etiologic agents of severe periodontitis [89]. Bacterial pathogens, which were the focus of this study, populate periodontal sites prior to a sequential infection by herpesviruses [2]. Both bacterial and herpesvirus pathogens inhabit deep periodontal lesions and inflamed gingiva [2], which tend to be beyond the reach of conventional surgical and non-surgical therapy and may require systemic anti-infective therapy. However, because of the wide range of periodontal pathogens, systemic antimicrobial therapy should optimally be based on a comprehensive microbiological analysis with in vitro antimicrobial susceptibility testing [4], as well as consideration of potential adverse side effects and drug interactions [22,35].

Two systemic antibiotic combinations in this study were assigned to specific patient categories based on microbiological testing of subgingival biofilms. Metronidazole-ciprofloxacin was prescribed for patients harboring both traditional anaerobic pathogens and various superinfecting enteric organisms, or positive for *A. actinomycetemcomitans* but hypersensitive to penicillin antibiotics, and where in vitro testing revealed susceptibility of the detected pathogens to either or both of the antibiotics. Metronidazole plus amoxicillin/clavulanic acid was prescribed to patients without a penicillin allergy history yielding subgingival *A. actinomycetemcomitans* or a mixed group of anaerobic and facultative periodontal pathogens demonstrating in vitro susceptibility to one or both of the antibiotics. For two patients, both combinations were sequentially prescribed to expand the antibacterial spectrum against a diverse array of traditional periodontal pathogens and superinfecting enteric rod species with varying antibiotic susceptibility profiles. 

“Refractory” periodontitis patients selectively treated with these antibiotic combinations experienced no further disease progression for at least 5 years, and exhibited gains in clinical periodontal attachment with significant decreases in probing depths, bleeding on probing, and subgingival temperature (Table 1). The greatest disease resolution occurred in patients where a high-risk subgingival microbiota was successfully suppressed and non-periodontopathic viridans streptococci markedly increased in subgingival biofilms (Table 5), which may serve to inhibit anaerobic bacterial recolonization and suppress pro-inflammatory host immune responses via their hydrogen peroxide production [90,91]. 

Local professionally-applied anti-infective therapy also contributed to these beneficial clinical and microbiological outcomes. Supragingival air-polishing used in this study during initial and supportive periodontal therapy effectively removes supragingival dental plaque [92] and, when carefully directed into subgingival sites, may also rapidly reduce bacterial populations in periodontal pockets [74]. Supragingival air-polishing may thus augment conventional mechanical tooth debridement procedures, which often leave residual dental plaque on supragingival tooth surfaces [93] and periodontal pathogens persisting in periodontal pockets [18,19,20]. Subgingival delivery in this study of a 0.1% povidone-iodine solution during ultrasonic scaler instrumentation provides significantly better clinical periodontal outcomes over a 12-year post-treatment period than conventional mechanical root debridement alone [78], largely due to better suppression of periodontal pathogens after adjunctive povidone-iodine disinfection of deep periodontal pockets [58].

Effective patient self-care is also critical to sustain a long-term benefit of the professionally administered anti-infective therapy [94]. Favorable microbiological changes induced by systemic periodontal antibiotic therapy are only short-lived in patients with flawed home oral hygiene [95,96]. However, mechanical removal of dental biofilms is faced with narrow interdental spaces and deep periodontal pockets, which may require application of antiseptics to remove the pathogenic biofilm on difficult-to-reach tooth surfaces. Oral irrigation devices enhance subgingival delivery of antimicrobial agent solutions in home care regimens [94], and 0.1% sodium hypochlorite was applied daily with oral irrigators by patients in the present study. Oral irrigation with dilute sodium hypochlorite, as compared to tap water, better inhibits dental plaque growth and prevents experimental gingivitis [97]. Dilute sodium hypochlorite is more active against Gram-negative periodontal pathogens than non-periodontopathic Gram-positive streptococci and *Actinomyces* species [98] and kills established dental plaque biofilms in vitro better than 0.1% chlorhexidine [98]. A 0.1% sodium hypochlorite solution exerts antimicrobial activity that exceeds average minimal inhibitory concentration (MIC) values for NaClO needed (ranging from 0.03–0.07%) against traditional periodontal pathogens *P. gingivalis*, *Prevotella intermedia*, *Fusobacterium nucleatum*, *Dialister pneumosintes*, and *A. actinomycetemcomitans*, and superinfecting species *Escherichia coli* and *Staphylococcus aureus*, and nearly for *Pseudomonas aeruginosa* (MIC = 0.13%) [99]. An almost complete turnover in the subgingival microbiome is found in severe periodontitis patients within 2 weeks and persists for at least 3 months after the start of twice-weekly rinsing with 0.25% sodium hypochlorite [100], with *P. gingivalis*, *T. forsythia*, *T. denticola* and other spirochete species no longer among the most abundant subgingival species [101], leading to markedly reduced bleeding in probing even in deep unscaled periodontal pockets [102]. The American Dental Association Council on Dental Therapeutics approved the use of dilute sodium hypochlorite solutions up to 0.25% for direct application onto human mucous membranes as a “mildly antiseptic mouthwash” [103]. An unpleasant taste of bleach is the most frequently reported side effect with intraoral use of dilute sodium hypochlorite solutions [100]. Oral rinsing and periodontal pocket irrigation with 0.1–0.2% sodium hypochlorite (dilute regular household bleach) is increasingly being used to combat periodontal bacteria and viruses, minimize aerosolization of infectious agents, and augment traditional oral hygiene measures [87,89]. 

The 65% sodium bicarbonate-based toothpaste used by patients in this study possesses high potential bactericidal activity against many putative periodontal bacterial pathogens [104,105,106] and *Streptococcus mutans* [107], is able to disaggregate established dental plaque biofilms in vitro [108], and provides better reductions in supragingival dental plaque growth and gingival bleeding than conventional non-baking soda toothpastes [109,110].

The present study was based on bacterial culture which allows for antimicrobial susceptibility testing but may have missed important pathogens identifiable by molecular methods [4]. Severe periodontitis lesions will typically harbor high levels of active herpesviruses [2], which can be detected by PCR and may require systemic anti-herpesvirus treatment [87,89]. Sunde et al. [111] treated Epstein-Barr virus-associated refractory periodontitis with 10 days of oral valacyclovir (500 mg twice daily). Posttreatment examination at 12 months revealed absence of periodontal Epstein–Barr virus and a “dramatically” improved periodontal health status. Sabeti et al. [112] treated symptomatic apical abscesses with 3 days of oral valacyclovir (2 g at baseline on day 1, and 500 mg, twice daily, on days 2 and 3). On the first day following baseline treatment, the valacyclovir group (*N* = 10) showed two patients with moderate pain and one patient on pain medication, whereas an amoxicillin “placebo” group (*N* = 10) revealed as many as eight patients with pain and nine patients on pain medication. These findings point to the validity of combining antibacterial antibiotics with anti-herpesvirus medication in the treatment of severe periodontitis. A proposed treatment outline for severe periodontitis was presented recently [87,89]. Future studies of periodontal therapy should encompass both antibacterial and anti-herpesvirus components.

## 5. Conclusions

Traditional treatment of severe periodontitis is burdened with high cost and uncertain therapeutic outcomes [87,89]. A complicating factor is the current therapeutic, almost exclusive, emphasis on mechanical control of bacterial biofilms and removal of dental calculus deposits, which is an appropriate sole therapy for simple gingivitis and mild periodontitis, but not for severe periodontitis. This patient case series demonstrates that a systemic anti-infective therapy targeting major periodontopathic bacteria, and preferably also active herpesviruses, combined with topical antimicrobials applied during non-surgical root debridement and daily patient home care, can achieve long-term disease resolution, even in patients who are refractory to conventional periodontal surgery. The personalized and minimally invasive anti-infective therapy described here is cost-effective as it involves fewer dental office visits and employs inexpensive generic medications. Low-cost periodontal therapy [113] may particularly benefit economically disadvantaged individuals, who are most affected by periodontitis and, without effective periodontal treatment, are at elevated risk of losing teeth and suffering systemic diseases from periodontal pathogens entering the systemic circulation and colonizing non-oral sites and organ systems.

## Figures and Tables

**Table 1:** Clinical parameters at baseline, and 1 and 5 years post-treatment.

	Treatment Group		
Clinical Parameter ^†^	I ^§^	II ^‡^	III ^‖^
No. of patients	23	10	2
Plaque Index scores:			
Baseline	0.6 (0.4)	0.5 (0.3)	0.6 (0.4)
1 year post-treatment	0.4 (0.3)	0.3 (0.2)	0.4 (0.3)
5 years post-treatment	0.4 (0.3)	0.3 (0.2)	0.4 (0.4)
% teeth with PD ≥ 6 mm:			
Baseline	39.0 (18.9)	29.6 (23.3)	40.8 (6.2)
1 year post-treatment	11.5 (9.9) *	8.9 (11.3) *	0
5 years post-treatment	9.6 (9.5) *	9.4 (13.8) *	0
% sites with BOP:			
Baseline	30.5 (18.1)	21.2 (13.8)	48.5 (16.3)
1 year post-treatment	2.5 (2.4) *	2.7 (3.3) *	1.7 (2.4)
5 years post-treatment	2.1 (1.8) *	1.7 (2.3) *	3.5 (4.9)
No. of sites with PD ≥ 5 mm and BOP:			
Baseline	33.3 (23.4)	29.0 (21.9)	22.0 (7.1)
1 year post-treatment	1.4 (2.4) *	2.0 (1.5) *	0.5 (0.7)
5 years post-treatment	1.2 (1.5) *	2.5 (2.3) *	0.5 (0.7)
**% sites with CAL gain > 2 mm:**			
1 year post-treatment	4.8 (4.6)	10.2 (10.7)	9.9 (0.2)
5 years post-treatment	5.9 (4.4)	6.3 (5.4)	6.8 (0.8)
**% sites with CAL loss > 2 mm:**			
1 year post-treatment	0	0	0
5 years post-treatment	0	0	0
Temperature indicator score	1.3 (0.4)	1.9 (0.6)	1.4 (0.2)
Subgingival temperature, °C	35.6 (0.7)	36.1 (0.5)	36.2 (0.4)
Subgingival–sublingual temperature differential, °C	−0.4 (0.6)	−0.7 (0.2)	−0.8 (0.3)

^†^ All values expressed as means per patient (SD), ^§^ received systemic metronidazole plus ciprofloxacin, ^‡^ received systemic metronidazole plus amoxicillin/clavulanic acid, ^‖^ received systemic metronidazole plus ciprofloxacin, and metronidazole plus amoxicillin/clavulanic acid, PD: probing depth, BOP: bleeding on probing, CAL: clinical periodontal attachment level, * Significantly different from baseline (*p* < 0.05).

**Table 2:** Microbiological parameters in Treatment Group I patients (N = 23).

		Post-Systemic Metronidazole Plus Ciprofloxacin	
Microbial Species	Baseline	1 Month Post-Treatment	1 Year Post-Treatment
Periodontal Pathogens:			
*Aggregatibacter actinomycetemcomitans*	6 (0.2) ^§^	0 ^§^	1 (0.1) ^§^
*Porphyromonas gingivalis*	5 (6.8)	0	1 (4.4)
*Prevotella intermedia/nigrescens*	9 (11.1)	4 (3.1)	4 (3.7)
*Parvimonas micra*	16 (12.0)	4 (2.4)	6 (8.6)
*Campylobacter rectus*	21 (3.7)	12 (0.6)	9 (1.1)
Superinfecting Species:			
enteric rods/pseudomonads	17 (20.3) ^§^	2 (0.5) ^§^	3 (0.5) ^§^
staphylococci	0	3 (1.4)	1 (1.1)
*Enterococcus faecalis*	0	0	0
*Candida* species	4 (0.1)	4 (3.4)	3 (0.8)
Mean % total recovery of test periodontal pathogens and superinfecting species (SD) ^†^	22.9 (18.2)	0.9 (1.7) *	2.4 (3.2) *
No. (%) of patients with high-risk subgingival microbiota ^‡^	23 (100)	3 (13.0) *	3 (13.0) *
Mean % recovery of viridans streptococci (SD)	2.6 (1.5)	24.2 (21.7) *	27.9 (22.2) *

^§^ No. of culture-positive patients (mean percent CFU recovery of test species among total subgingival viable counts in species-positive patients), ^†^ Total cultivable subgingival proportions of the selected species among total viable subgingival anaerobic counts per patient, ^‡^ Defined as cultivable presence of either *A. actinomycetemcomitans* and/or *P. gingivalis*, and/or cultivable recovery of *P. intermedia/nigrescens* at ≥2.5%, *P. micra* at ≥3.0%, *C. rectus* at ≥2.0%, and/or either Gram-negative enteric rods/pseudomonads, staphylococci, or *Candida* species at ≥5.0%, of total subgingival viable counts, * Significantly different from baseline (*p* < 0.05).

**Table 3:** Microbiological parameters in Treatment Group II patients (N = 10).

		Post-Systemic Metronidazole Plus Amoxicillin/Clavulanic Acid	
Microbial Species	Baseline	1 Month Post-Treatment	1 Year Post-Treatment
**Periodontal Pathogens:**			
*Aggregatibacter actinomycetemcomitans*	7 (2.9) ^§^	0 ^§^	1 (0.01) ^§^
*Porphyromonas gingivalis*	1 (14.3)	0	0
*Prevotella intermedia/nigrescens*	8 (6.2)	0	3 (2.3)
*Parvimonas micra*	7 (9.6)	4 (3.2)	1 (21.6)
*Campylobacter rectus*	9 (2.7)	4 (0.4)	4 (2.5)
Superinfecting Species:			
enteric rods/pseudomonads	1 (0.5) ^§^	2 (18.7) ^§^	2 (7.2) ^§^
staphylococci	0	0	1 (2.7)
*Enterococcus faecalis*	0	0	0
*Candida* species	0	2 (0.03)	3 (0.02)
Mean % total recovery of test periodontal pathogens and superinfecting species (SD) ^†^	18.2 (10.6)	1.3 (2.4) *	3.5 (3.1) *
No. (%) of patients with high-risk subgingival microbiota ^‡^	10 (100)	2 (20.0) *	4 (40.0) *
Mean % recovery of viridans streptococci (SD)	5.7 (3.4)	18.5 (14.4) *	19.1 (22.7) *

See Table 2 footnote for symbol definitions.

**Table 4:** Microbiological parameters in Treatment Group III patients (N = 2).

		Post-Systemic Metronidazole Plus Ciprofloxacin, and Metronidazole Plus Amoxicillin/Clavulanic Acid	
Microbial Species	Baseline	1 Month Post-Treatment	1 Year Post-Treatment
Periodontal Pathogens:			
*Aggregatibacter actinomycetemcomitans*	2 (7.2) ^§^	0 ^§^	0 ^§^
*Porphyromonas gingivalis*	0	0	0
*Prevotella intermedia/nigrescens*	2 (6.8)	0	0
*Parvimonas micra*	2 (7.4)	1 (1.6)	0
*Campylobacter rectus*	2 (18.7)	1 (0.1)	1 (1.3)
Superinfecting Species:			
enteric rods/pseudomonads	2 (19.7) ^§^	0 **^§^**	0 ^§^
staphylococci	0	0	0
*Enterococcus faecalis*	0	0	0
*Candida* species	0	0	1 (0.01)
Mean % total recovery of test periodontal pathogens and superinfecting species (SD) ^†^	50.7 (19.9)	0.9 (1.1)	0.7 (0.9)
No. (%) of patients with high-risk subgingival microbiota ^‡^	2 (100)	0 (0)	0 (0)
Mean % recovery of viridans streptococci (SD)	6.5 (5.7)	16.6 (8.4)	31.0 (19.0)

See Table 2 footnote for symbol definitions.

**Table 5:** Relationship between high-risk subgingival microbiota and clinical periodontal disease resolution.

		Mean No. of Periodontal Sites/Patient with PD ≥ 5 mm and BOP (SD)	
High-Risk Subgingival Microbiota ^‡^ Detected at 1 Month and/or 1 Year Post-Treatment	No. of Patients	1 Year Post-Treatment	5 Years Post-Treatment
Yes	12	3.2 (2.8)	2.9 (2.1)
No	23	0.7 (0.9) *	0.8 (1.1) *

^‡^ Defined as cultivable presence of either *A. actinomycetemcomitans* and/or *P. gingivalis*, and/or cultivable recovery of *P. intermedia/nigrescens* at ≥2.5%, *P. micra* at ≥3.0%, *C. rectus* at ≥2.0%, and/or either Gram-negative enteric rods/pseudomonads, staphylococci, or *Candida* species at ≥5.0%, of total subgingival viable counts, PD: probing depth, BOP: bleeding on probing, * Significantly different from positive patients (*p* < 0.05).

## Data Availability

Not applicable.
